# Biofilm Bridges Forming Structural Networks on Patterned Lubricant‐Infused Surfaces

**DOI:** 10.1002/advs.201900519

**Published:** 2019-05-08

**Authors:** Wenxi Lei, Julia Bruchmann, Jan Lars Rüping, Pavel A. Levkin, Thomas Schwartz

**Affiliations:** ^1^ Institute of Toxicology and Genetics Karlsruhe Institute of Technology Hermann‐von‐Helmholtz Platz 1 76344 Eggenstein‐Leopoldshafen Germany; ^2^ Institute of Functional Interfaces Karlsruhe Institute of Technology Hermann‐von‐Helmholtz Platz 1 76344 Eggenstein‐Leopoldshafen Germany; ^3^ Institute of Organic Chemistry Karlsruhe Institute of Technology 76131 Karlsruhe Germany

**Keywords:** antibiofouling, biofilm bridging, biofilm structure, liquid‐infused surfaces, networks, patterned “slippery” lubricant‐infused porous surfaces (SLIPS)

## Abstract

Despite many decades of research, biofilm architecture and spreading mechanisms are still not clear because of the heterogenous 3D structure within biofilms. Here, patterned “slippery” lubricant‐infused porous surfaces are utilized to study biofilm structure of *Pseudomonas aeruginosa*, *Stenotrophomonas maltophilia*, and *Staphylococcus aureus*. It is found that bacteria are able to spread over bacteria‐repellent lubricant‐infused regions by using a mechanism, termed “biofilm bridges”. Here, it is demonstrated that bacteria use bridges to form interconnected networks between distant biofilm colonies. Detailed structure of bridges shows a spatial distribution of bacteria with an accumulation of respiratory active bacteria and biomass in the bridges. The core–shell structure of bridges formed by two‐species mixed population is illustrated. It is demonstrated that eDNA and nutrients have a strong effect on biofilm bridges formation. Thus, it is believed that biofilm bridging is important to reveal the structure and communication within biofilms.

## 1 Introduction

Biofilms on surfaces is the predominant form of bacterial lifestyle not only in technical settings and nature but also in 80% of all infections in medicine.^1^ Such sessile bacterial communities work as a team through varied interaction and communication such as horizontal gene transfer, protein exchange, and quorum sensing (QS).^2^, ^3^ Despite a lot of research, internal organization, interactions within biofilms, mechanics and details behind biofilm development often remain to be determined. Reasons for this lie in the heterogeneity of biofilms, which leads to high variances in the gene expression, stress response, and behavior of different subpopulations.^4^ The lack of understanding of biofilm spreading is especially important in clinical settings, where the host immune system, drug administration, or other factors can influence biofilm expansion and may result in severe conditions.^5^ Furthermore, biofilm removal or manipulation is a major cost intensive factor in technical systems as high consumptions of toxic biocides or mechanical efforts are performed to avoid biofilm formation (water condition and distribution). Biofilms play a significant role in medicine since high numbers of infections originate from biofilm contaminations, e.g., at implants. These biofilms are much more insensitive against antibiotics than planktonic pathogens especially in case of multiresistance against antibiotic drugs. Hence, there is an urgent need to design models aiding us to investigate structure, interconnectivity, diversity, and dynamics in biofilm in a controllable way.

Biofilms are highly heterogenous due to their spatial partitions in larger structures (landscape), which leads to the inability to look into fine structural changes of biofilm communities as a function of various relevant factors. Hence, fine changes, which are often critical in understanding structure–function relationship in biofilms, are often overlooked in case of such bulk analyses. In addition, every laboratory uses a different method for biofilm investigations, which might have a significant influence on biofilm behavior, e.g., medium composition, construction of flow cells, fluidic versus static culturing, etc. Biofilm cannot be considered as a simple sum of individual bacterial cells, but as a complex differentiated community with a heterogenous 3D structure.^6^ Biofilms represent organized communities encased in a matrix of extracellular polymeric substances (EPS) that hold microbial cells together to a surface.^7^ EPS is composed mainly of biomolecules, exopolysaccharides, extracellular DNA (eDNA), and polypeptides that form a highly hydrated polar mixture that contributes to the overall structural scaffold and architecture of the biofilm.^6^ Depending on the bacterial species or strains and the nutritional conditions, different biofilm phenotypes can be developed starting with a reversible attachment to surface, followed by irreversible colonization with formation of micro‐colonies in EPS‐matrix. Bacterial micro‐colonies expand and a more structured phenotype with channels and voids is developed during biofilm maturation. Finally, bacteria disperse from biofilm structures and spread to downstream areas forming new biofilms. One of the special structural assemblies in biofilm are biofilm streamers, which occur under flow conditions along the fluidic direction.^8^ These filamentous structural streamers of, e.g., *Pseudomonas aeruginosa* are networks of biofilm filaments consisting of EPS and bacteria. By catching cells flowing through the gaps between them, streamers are able to connect bacterial clusters and promote spreading of biofilm.^9^ Revealing structure–function relationship in biofilms might help us to prevent biofilm spreading and invasion in all kinds of medical and technical system. However, suitable assays for analysis of biofilm spreading are still missing.

Bioinspired “slippery” lubricant‐infused porous surfaces (SLIPS) have been exploited in various applications, including prevention of eukaryotic cell and biofilm adhesion.^10^ Due to the liquid‐like properties and the defect‐free nature of SLIPS, it is difficult for mammalian cells and bacteria to attach onto them irreversibly.^11^ It is reported that SLIPS were able to decrease the biofilm occupation on surfaces.^12^ Recently, we demonstrated a method to form arrays of biofilm clusters with defined 2D geometries by using patterned SLIPS. To our surprise, on lubricant‐infused bacteria repellent regions, biofilm bridges were formed spontaneously between neighboring clusters of *Pseudomonas aeruginosa* separated by SLIPS regions in the range of 50–500 µm.^13^ We hypothesized that bacteria used this phenomenon to expand beyond adhesive hydrophilic spots more efficiently.

Here, we apply patterned SLIPS to create spatially separated biofilm clusters to investigate the phenomenon of biofilm bridging. Patterned SLIPS is a useful tool to study biofilm bridging, as it builds up physical “walls” between biofilm clusters, while allowing transport of signals, nutrients, and bacteria between them. We used both Gram‐negative species including *Pseudomonas aeruginosa*, *Stenotrophomonas maltophilia* and Gram‐positive species *Staphylococcus aureus* to investigate biofilm bridges. Fine structure of bridges and metabolic activity were studied with fluorescence microscopy. Fluorescence in situ hybridization (FISH) demonstrated the structural organization of bridges consisting of two‐species mixed populations. Network of biofilm bridges was investigated. Further, possible factors that influence bridging such as the role of eDNA in biofilm and nutrients were investigated.

## 2 Results and Discussion

### 2.1 Formation of Biofilm Bridges of *P. aeruginosa* PA 49 over SLIPS

**Figure 1:**
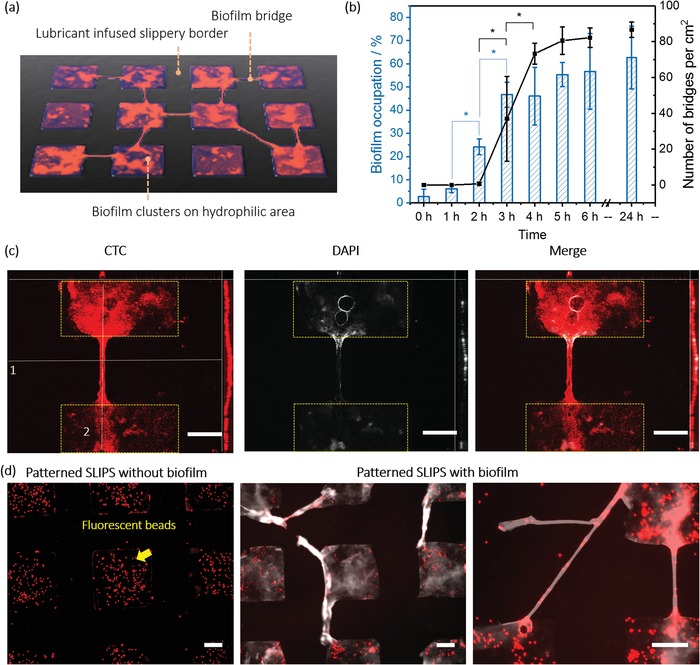
a) Scheme of biofilm bridges formation of *P. aeruginosa* PA 49 on patterned SLIPS. b) Numbers of bridges and biofilm occupation of *P. aeruginosa* PA 49 on patterned SLIPS after certain time point in bacterial suspension of BM2 medium. DAPI staining coverage of hydrophilic area calculated from fluorescence images with ImageJ software is presented as biofilm occupation. c) Z‐stack images of biofilm bridges. (Left: CTC staining. Middle: DAPI staining. Right: merge. For each image, up: cross‐section, corresponding line1; right: cross‐section, corresponding line 2, middle: top view). Patterned SLIPS were incubated with *P. aeruginosa* PA 49 for 24 h. Then samples were stained with CTC (red color) and DAPI (white color). The thickness of the Z‐stack is 40 µm. d) Fluorescence microscope images of patterned SLIPS after deposition of microbeads labeled with red dye: (left) without biofilm and (right two) with *P. aeruginosa* PA 49 biofilm formed during 24 h. Both samples were stained with DAPI (white color), followed by 10 min incubation with the microbeads (1 µm) for 10 min and washing with water. The scale bars: 100 µm.

In order to investigate the phenomenon of biofilm bridges, we first formed an array of bacteria adhesive hydrophilic squares with side length of 350 µm separated by 200 µm lubricant‐infused biofilm repellent regions. Perfluorinated polypropyleneoxide (Krytox GPL 103) was used as the lubricant. Scanning electron microscope image shows the porous structure of the surface (Figure S1, Supporting Information), which is required to lock lubricant and form a stable lubricant layer. Water contact angles and sliding angles of patterned surfaces with and without lubricant were shown in Table S1 in the Supporting Information. The sliding angles of lubricant‐infused surfaces were 1.6°± 0.2°, while the advancing water contact angles were 100.4°± 5° and receding water contact angles were 95.5°± 2°, indicating the slippery property of the surfaces. Patterned slides were incubated in *P. aeruginosa* PA 49 strain suspension under shaking to grow biofilm clusters on hydrophilic spots (**Figure** **1**a). We used 4',6‐diamidino‐2‐phenylindole (DAPI) to stain both intracellular DNA in bacteria and DNA in EPS of biofilms. The first biofilm bridges were observed on surfaces after 3 h incubation (Figure 1b), which increased up to 82.2 ± 5.4 bridges per cm^2^ after 6 h of incubation. Each hydrophilic square showed 0.24 biofilm bridges on average after 24 h incubation. The occupation of biofilm in hydrophilic spots increased with longer incubation time as well, from 6.1 ± 1.6% after 1 h incubation to more than 56.6 ± 16.3% of the hydrophilic area of each cluster after 6 h incubation. These observations suggested that the biofilms were formed on the hydrophilic spots already after 1 h of incubation, while the first bridges were detected only after 3 h. String‐like structure of biofilm was demonstrated before. Jahed et al. used dewetting properties of poly(dimethyl siloxane) micropillars to fabricate “biostrings” of *S. aureus* after liquid retracting process.^14^ Different from this approach, the biofilm bridges formed in our study were not caused by fluid mechanics, as biofilm bridges were observed on patterned SLIPS incubated under both static and static with shaking condition (Figure S2, Supporting Information).

Based on the fluorescence images in Figure S7 in the Supporting Information, we can hypothesize the following steps for bridge formation (Figure S6, Supporting Information): 1) the bacteria attach to the hydrophilic area. During this period, the hydrophilic areas are covered by biofilm gradually. 2) Biofilms form in the hydrophilic areas during 2 h incubation. Biomass of biofilm reaches a threshold. 3) Bridges start to form. One possibility is that bacteria grow from the one side of the SLIPS barrier to reach the cluster on the other side of the barrier (see Figure S7, images 8, 11, 14, 17, 23, 29, Supporting Information). We also see incomplete biofilm bridges that start on both sides of the SLIPS region but seem to grow toward each other (see Figure S7, images 5, 20, Supporting Information).

To demonstrate the scale of the bridge structure, we analyzed Z‐stack images of the bridge of *P. aeruginosa* PA 49 after 24 h incubation in BM2 medium. As shown in Figure 1c, the bridge did not attach to the substrate surface such as the biofilm grown in hydrophilic spots but rather formed an arc above the substrate's plane. The distance between the highest part of the bridge and the substrate was 20.4 µm. This distance should be caused by the existence of lubricant, making the SLIPS plane higher than that of the hydrophilic area. To prove this further, we used 1 µm microbeads labeled with red dye to incubate with patterned SLIPS with and without *P. aeruginosa* PA 49 biofilm formed. All samples were incubated in BM2 medium for 24 h, stained with DAPI and incubated with the fluorescent beads for 10 min. As shown in Figure 1d (left), on the patterned SLIPS without bacteria, the beads only aggregate in the hydrophilic spots, suggesting that beads tend to sediment and bind to the hydrophilic areas. Figure 1d (right) shows that there was overlay of beads and bridges, confirming that bridges were exposed to the medium enabling their interaction with the fluorescent beads. The specificity of this microbead attachment to biofilm and bridge structures illustrates that the bridges are located on top of the lubricant area and not covered by the oil.

### 2.2 Formation of Biofilm Bridges is a Ubiquitous Phenomenon Among Different Bacteria Species

In order to understand whether the biofilm bridge formation is a ubiquitous phenomenon during biofilm growth in the bacterial world, four different bacterial species were selected. Two strains of *P. aeruginosa*, PA 30 and PA 49, as well as *S. maltophilia* were used as Gram‐negative species, which occur in lung infection and urinary tract infection.^15^, ^16^ In addition, we used *S. aureus*, a Gram‐positive pathogenic bacteria involved in broad clinical infections such as infective endocarditis and osteoarthritis.^17^ These facultative‐pathogenic bacteria are frequently associated with nosocomial infections and tend to form multiresistances against clinically relevant antibiotics, which can hardly become medically treated in case of infections.^18^

**Figure 2:**
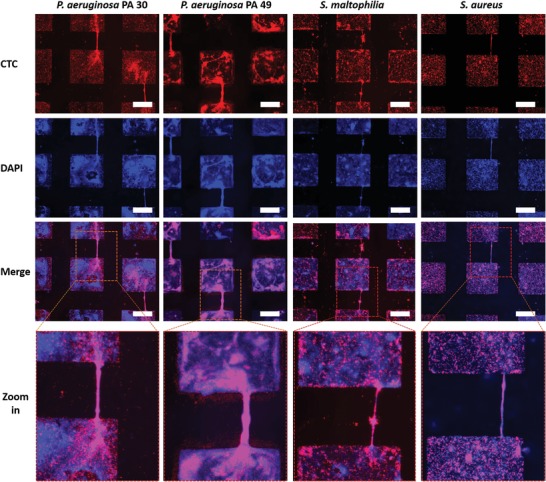
Fluorescence microscope images of biofilms of different species on patterned SLIPS. *P. aeruginosa* PA 30, PA 49, *S. maltophilia*, and *S. aureus* after 1 day incubation in BHI 1:4 medium. Biofilms were stained with CTC for 3 h then with DAPI for 10 min before images were produced. Red color represents active bacteria from CTC staining and blue color represents DNA (external + inside of bacteria). The microscope observations were completed by ImageJ software. The scale bar is 200 µm.

After incubation with bacterial solution for 24 h, SLIPS samples were removed from the petri dishes and stained with 5‐cyano‐2,3‐ditolyl tetrazolium chloride (CTC) and DAPI. As shown in **Figure** **2**, for all species biofilms were formed on hydrophilic areas. Actively respiring, CTC‐positive bacteria (red fluorescence) could be observed in hydrophilic squares and only a few attached aggregates of bacteria were detected on hydrophobic slippery areas. The blue fluorescence from DAPI, staining intra‐ and extracellular DNA, was also found predominantly in the hydrophilic bacteria‐adhesive squares with only a few biofilm colonies in the lubricant‐infused regions. The biofilm bridges were clearly observed for all species under investigation (Figure 2). Biofilm bridges represented thin biofilm strings showing active metabolism (CTC‐positive) and presence of intra and extracellular DNA (DAPI‐positive) and connecting adjacent biofilm clusters formed in the hydrophilic squares. Interestingly, the CTC‐staining of the bridges was brighter than that of the biofilm main clusters, indicating presence of highly active bacteria in the bridges (see also Figure 4). The shape of the bridges depended on the bacteria strain. For *P. aeruginosa* PA30 and PA49, the bridges were dense, uniform, with bright fluorescence of respiring bacteria, and total DNA, indicating a possible interaction of active bacteria and EPS. Bridge formation by *S. maltophilia* performed differently compared to the other two bacteria types. This indicates that bridge formation is species dependent.

**Figure 3:**
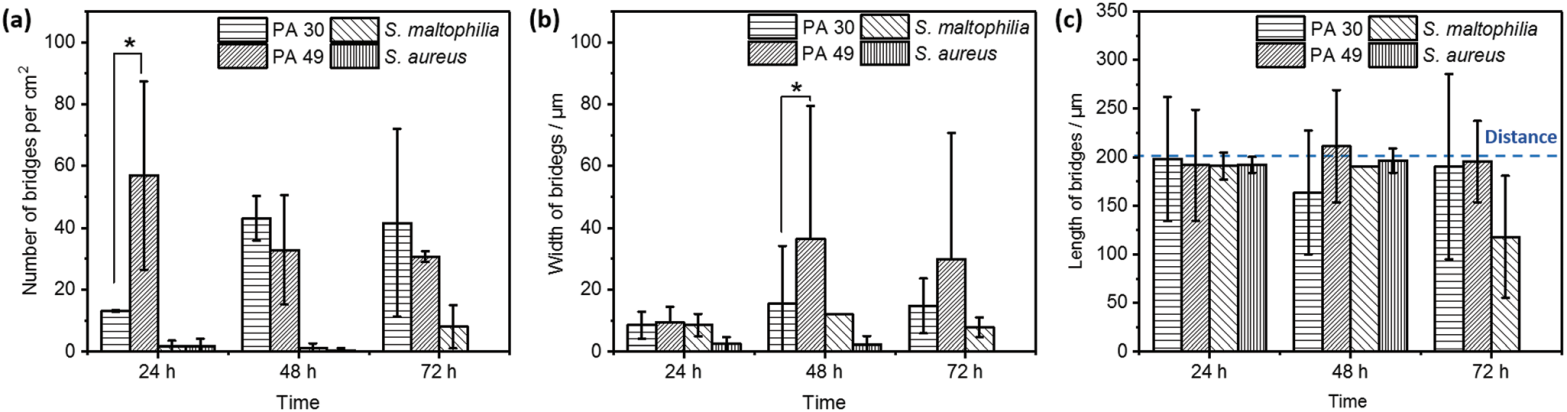
Formation of biofilm bridges for *P. aeruginosa* (PA 30, PA 49), *S. maltophilia*, and *S. aureus*. a) Number of bridges per area (cm^2^), b) width of bridges at the middle of a bridge, and c) length of bridges. Biofilm bridges were analyzed on patterned SLIPS after 1, 2, and 3 days incubation in BHI 1:4 medium and stained with CTC and DAPI. The dotted line in (c) represents the closest distance between neighboring hydrophilic spots (200 µm).

**Figure** **3**a shows the time‐dependent formation of biofilm bridges for all species studied. The density of bridges for *P. aeruginosa* PA49 was the highest among all species. It was 56.9 ± 30.4 bridges per cm^2^ (0.2 ± 0.1 bridges per hydrophilic square), which is almost four times more than *P. aeruginosa* PA 30 (13.2 ± 0.3 bridges per cm^2^_,_ 0.1 bridges per a hydrophilic square) after 24 h incubation. *S. maltophilia* and *S. aureus* developed only 1.7 ± 1.6 and 1.8 ± 2.2 bridges per cm^2^, respectively (0.01 and 0.01 bridges per a hydrophilic square, respectively), much less than the both *P. aeruginosa* strains PA30 and PA 49. The number of bridges increased for *P. aeruginosa* PA30 to 43.1 ± 7.9 bridges per cm^2^ after 48 h with refreshing the nutrient medium after 24 h. This increase was not observed with *P. aeruginosa* PA49 with similar experimental conditions. Similar results were obtained with *S. maltophilia* and *S. aureus* with an unchanged number of bridges after 24 h incubation. More generally, the density of bridges of all species did not increase significantly after 48 h incubation.

The width of bridges ranged from few micrometers to more than 70 µm, depending on the species and incubation times (Figure 3b). The width of bridges changed with the incubation time especially for *P. aeruginosa* PA 49, which increased from 9.4 ± 5.0 to 36.4 µm and the broadest bridges could reach 79.4 µm after 48 h incubation. Nevertheless, this increase did not continue in the next 24 h incubation. For *P. aeruginosa* PA 30, the width of bridges increased from 8.5 ± 4.1 µm after 24 h incubation to the broadest 34.1 µm after 48 h incubation. There were no obvious changes in width of bridges for other species with time, as most were in a range of dimension from 2 to 20 µm. The distance between hydrophilic squares being 200 µm, therefore the length of bridges for all bacterial species was around 200 µm. For half bridges, which were connected with only one biofilm cluster, the length was shorter than 200 µm (Figure 3c). In some cases, biofilm bridges longer than the side‐to‐side distance between hydrophilic squares were observed. For example, connecting two corners from two biofilm squares diagonally resulted in bridges of around 280 µm. As previously described, *P. aeruginosa* PA 49 is known to possess an increased biofilm formation capacity compared to *P. aeruginosa* PA 30.^19^ This higher biofilm‐forming potential could contribute to the increased bridge development especially during the first 24 h of incubation. Either the increase of bridge width of *P. aeruginosa* PA 49 could be responsible for a possible start of biofilm spreading on the biofilm repellent slippery area. Bridge formation by *S. maltophilia* performed differently comparing to the other two bacteria types which indicates that bridge formation is species dependent.

### 2.3 Biofilm Bridges' Composition and Structure

**Figure 4:**
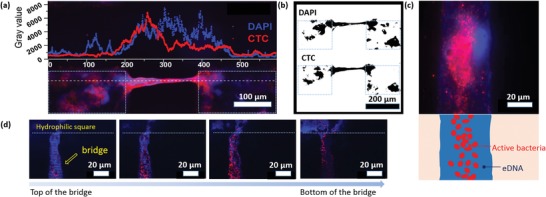
Fluorescence microscope images of bridges of *P. aeruginosa* PA 49 after 24 h biofilm formation stained with CTC (metabolically active cells, red) and DAPI (DNA as total biomass indicator, blue). a) Fluorescence intensity of CTC and DAPI staining of the line (white dot line) along a biofilm bridge connecting two biofilm clusters. b) Images of the biofilm bridge after threshold adjustment. c) Fluorescence microscope image of a biofilm bridge at a higher magnification showing active bacteria and extracellular structural DNA (top). Corresponding schematic (bottom). d) Z‐stack images of a biofilm bridge. Images from left to right represent features of bridges at different *Z*‐positions from the top to the bottom of the bridge.

To investigate the structure and composition of biofilm bridges, high magnification fluorescence microscopy was used. **Figure** **4**a shows both CTC and DAPI fluorescence intensities plots along a single *P. aeruginosa* PA 49 biofilm bridge formed after 24 h incubation. The fluorescence intensity corresponding to the metabolically active bacteria in the bridge (5000–6000 gray unit) was about three times higher than the fluorescence intensity of the biofilm located in the neighboring hydrophilic spots (1500–2000 gray unit). Interestingly, both the CTC and DAPI fluorescence increased not only in the bridge but also in the areas adjacent to the ends of bridges, where bridges attached to the main biofilm clusters (Figure 4a,b). Such bright fluorescence demonstrated an aggregation of actively respiring bacteria and eDNA in the bridge structures including the attachment points of the bridges. There is a clear overlap of both signals indicating the co‐existence of active bacteria and eDNA inside the bridge.

Figure 4c shows respiratory active bacteria were surrounded with a layer of nucleic acids (eDNA) as part of the EPS or nonactive bacteria. Z‐stacks scanning was also used to analyze the bridges in more detail. Figure 4d shows from the top of the bridge, fluorescence from DAPI staining was first presented, revealing that it is eDNA components of the EPS but not respiratory active bacteria exposed directly to environment. Such structure was described in Figure 4c. As commonly known, EPS plays a critical role in biofilm formation and contributes to some crucial features of biofilms, such as antibiotic‐resistance, high tolerance of environmental stress, and difficult eradication in biofilm bridges, EPS occurs as a protective shell for inner respiratory active bacteria, indicating the role of EPS is necessary for biofilm bridges formation and stability.^20^

### 2.4 eDNA Stabilizes Biofilm Bridges of *P. aeruginosa* and *S. maltophilia* on Patterned SLIPS

**Figure 5:**
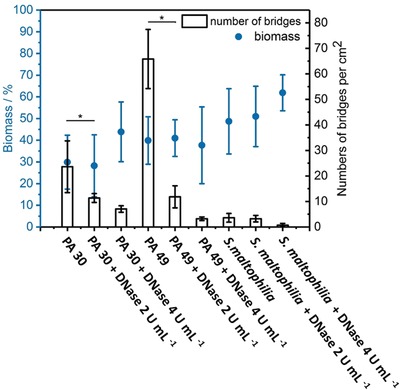
Number of bridges and coverage of biomass of *P. aeruginosa* PA 30, PA 49 and *S. maltophilia* after 1 day incubation in BHI medium with different dose of DNAse added.

DNase was added to bacterial suspension (*P. aeruginosa* PA 30, PA 49, *S. maltophilia*) from the beginning of incubation of bacteria on SLIPS patterns. The number of *P. aeruginosa* PA 30 bridges decreased from 23.6 bridges per cm^2^ on control slides (without DNase) to 11.3 bridges per cm^2^ and to 7.1 bridges per cm^2^ in the presence of 2 and 4 U mL^−1^ DNase after 24 h incubation. Similarly, the number of *P. aeruginosa* PA 49 bridges decreased from 65.8 to 11.8 bridges per cm^2^ and 3.2 bridges per cm^2^ after the addition of 2 and 4 U mL^−1^ DNase, respectively. The number of *S. maltophilia* bridges also decreased from 3.6 to 0.1 bridges per cm^2^ with 4 U mL^−1^ DNase in incubation medium (**Figure** **5**). However, the biomass coverage did not decrease significantly for all species (all biomass coverage fluctuated in a range of 25–70%, Figure 5), suggesting the growth of biofilms on hydrophilic area was not influenced apparently. eDNA in biofilm EPS plays a crucial multifunctional role in exchange of genetic information, structural integrity maintaining, horizontal gene transfer, and protection of biofilms from antibiotics or the host immune system.^21^ Specifically, eDNA facilitates twitching motility of bacteria by directing the traffic flow of cells to the leading edges of biofilm for further migration, and is therefore relevant for the biofilm spreading.^3^ The decreased number of bridges suggested eDNA is also essential in bridging process, for it could support communication between biofilm clusters and construct the EPS‐protecting structure shown in Figure 4c.

### 2.5 Mixed Species Biofilm Bridges

**Figure 6:**
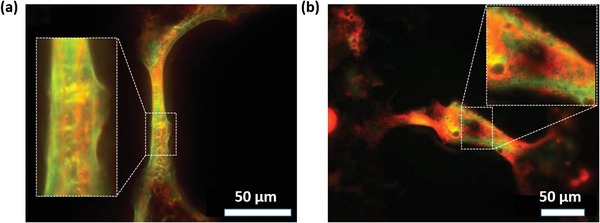
Images of biofilm bridges of mixed species of a) *S. maltophilia* and *P. aeruginosa* PA 49. b) *S. maltophilia* and *P. aeruginosa* PA 30 after FISH hybridization.

Here, we used FISH analysis with the fluorescently labeled gene probes targeting specific sequences of the 16S rRNA of different bacteria types cultured on patterned SLIPS samples, aiming to deeper understand the composition of bridges and the spatial distribution of the cells in bridges. The oligo‐nucleotide for *S. maltophilia* was labeled with a red fluorescence dye (ATTO550), whereas *P. aeruginosa* PA 30 and PA 49 were labeled with a green fluorescence dye (AT488). Although both bacterial species were found in the same biofilm bridge, the fluorescence images in **Figure** **6**a,b and Figure S3 in the Supporting Information show a spatial segregation of the two different investigated bacterial species in the biofilm bridges. The two bacteria types in the bridges did not mix homogenously but at the same time utilized this structural element of the biofilm. Both red and green fluorescent “strings” corresponding to each bacterial species were few micrometers thick and went along the whole length of the bridge, which was clearly visible inside individual mixed population biofilm bridges (Figure 6). This observation may indicate the importance of the bridges as a functional unit of biofilm and shows the use of such elements by different bacterial species together, which in turn may be beneficial for the overall survival of biofilms.

To the best of our knowledge, this core–shell structure was not reported before. The co‐existence of *P. aeruginosa* and *S. maltophilia* in the biofilm bridges would be the result of coaggregation interaction, which is caused by protein adhesions on bacteria surfaces, and other structural biofilm relevant factors.^22^ The segregation and specific spatial organization of *P. aeruginosa* and *S. maltophilia* in the bridges are one of typical characteristics of co‐operative interactions in multiple species biofilms, which is beneficial for efficient diffusion path for organic compounds such as nutrients and signaling molecules.^22^

### 2.6 Factors Influencing Networks of Biofilm Bridges of PA 49

**Figure 7:**
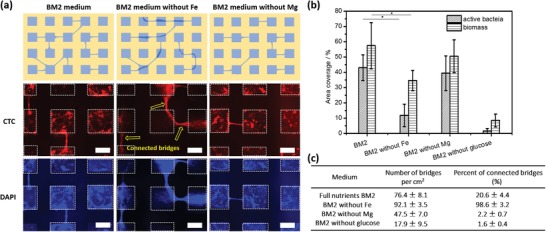
The effect of nutrients on biofilm bridges. PA49 was cultured for 1 day in original BM2 medium, BM2 medium without Fe, BM2 medium without Mg, and BM2 medium without glucose. a) Schematic illustration of different types of networks of biofilm bridges depending on the presence or absence of Fe and Mg in the medium. (Below) Fluorescence images of biofilm bridges stained with CTC and DAPI. Square hydrophilic spots are indicated by white dashed lines. The scale bar is 200 µm. b) Area coverage (in %) of metabolically active bacteria (stained with CTC) and eDNA (stained with DAPI) after 1 day incubation under different conditions. The graph shows the importance of Fe and glucose present in the medium for the amount of active bacteria on the surface. c) Number of biofilm bridges per cm^2^ and the percentage of bridges where at least one end of the bridge is connected with another bridge through the biofilm cluster formed in the hydrophilic square (number of “connected bridges”).

Biofilm formation, growth, and spreading are affected by the concentration as well as composition of nutrients in media. The effect of magnesium ions on biofilm formation is complex and is dependent on the stages of biofilm formation.^23^ For some species, during initial stage of biofilm formation, magnesium ions are required for promoting the attachment of planktonic bacteria on surfaces.^24^ For later stage, magnesium ions are crucial for the buildup of heterogenous biofilm structure.^23^ Iron ions are known to play an important role in QS.^25^ What is more, lack of iron would enhance the motility of bacteria, inhibiting the settlement of bacteria on surface.^26^ Considering bridging is a part of biofilm development on SLIPS, we assumed the nutrients might have an impact on bridge formation. In order to investigate this hypothesis, *P. aeruginosa* PA 49 was incubated on SLIPS patterns in mineral BM2 medium, BM2 medium without magnesium ions, and BM2 medium without iron ions and analyzed the effect of the medium composition on biofilm bridging. As shown in **Figure** **7**b, biofilms on SLIPS in BM2 medium without iron, the eDNA as part of the EPS and the respiratory active bacteria were four times less compared to SLIPS in original medium, as only very weak red fluorescence and blue fluorescence was observed in the squares except for the bridges. However, the number of bridges was higher than bridges in control medium (Figure 7c). Most analyzed bridges were connected to each other through the squares (98.6 ± 3.2%), which was not observed for bridges in original medium (Figure 7a, see yellow arrow). Iron limitation was able to induce the production of the biosurfactant rhamnolipid in *P. aeruginosa*. Therefore, the elevated concentration of the rhamnolipids may increase the motility of the biofilm bacteria and therefore support the expansion of biofilms under stress conditions (here: iron limitation).^27^ We hypothesized that under the pressure of iron insufficiency, bacteria tended to build up more bridges and grow along the bridges to explore larger territory instead of settling on hydrophilic squares, meaning that the bridges formation would be considered as one parameter of stress response of biofilm. On the other hand, the shortage of iron ions benefits DNA release from bacteria cells to biofilm EPS.^28^ The increased amount of eDNA would support the development of biofilm bridges as we described in Section 2.4. Hence, intense biofilm networking, which would contribute to long‐term exchange of, e.g., signal molecules, is a helpful tool that reacts on manipulated environmental milieus. As iron ions are only an example to manipulate the environmental conditions, more relevant technical or medical factors should be studied in the future.

The bridges formed on SLIPS in medium without magnesium ions showed less number (47 ± 7 bridges per cm^2^) than the bridges (76 ± 8 bridges per cm^2^) on SLIPS in original BM2 medium (Figure 7c). While the coverage of active respiring bacteria and biomass (DNA) was not decreased so much (Figure 7b). These results suggest a facilitation effect of magnesium ions on bridges formation. In addition, for patterned SLIPS in medium without glucose, the formation of biofilms and bridges was inhibited apparently. Because weak fluorescence of DAPI staining and CTC staining was observed (biofilm coverage ≤ 10%, Figure 7b). However, bridges could still form though the number of bridges was much less compared to those on SLIPS in the original medium. Such phenomenon means that it was possible to grow biofilm bridges even without strong biofilm structure as background.

## 3 Conclusion

Microcluster analyses are fundamental in studying biofilm structures and stimuli‐dependent reactions of biofilms. Here we describe a novel important phenomenon: the biofilm bridging. This phenomenon might have implications in biofilm development, spreading, and surpassing adverse surface conditions. On patterned SLIPS, bacteria did not restrictedly grow in hydrophilic regions but spread over bacteria‐repulsive regions and contacted with other biofilm clusters with thin biofilm bridges. It was shown that this bridging behavior is common to different Gram‐negative and Gram‐positive bacteria in species‐dependent manner. Organisms' distribution and organization in bridges of multispecies biofilm were demonstrated. The formation of biofilm bridges is important to bring deeper understanding of biofilm complex 3D structure. We further investigated the shown importance of eDNA and nutrient availability on biofilm bridges. In particular, bridges formation acted as a stress response with limited iron ion availability. By manipulating incubation environment, formation of networks composed of bridges between biofilm clusters and spreading over multiple biofilm clusters were discovered. Thus, biofilm bridge formation is an important novel phenomenon, which can be useful to reveal more details about the dynamics and communication within biofilm communities as well as to understand the relations of subpopulations, stress responses including virulence regulations, and biofilm spreading.

## 4 Experimental Section

*Materials and Instruments*: Patterned superhydrophobic–hydrophilic patterned glass slides (7.5 × 2.5 cm) were obtained from Aquarray GmbH (Germany). Each slide had three compartments and each compartment had 39 × 39 square‐shape hydrophilic spots. The size of each spot is 350 × 350 µm. The distance between hydrophilic spots was 200 µm. Ethanol was purchased from Merck (Darmstadt, Germany). Krytox GPL 103 (Dupont KrytoxR GPL 103) was purchased from H Costenoble GmbH & Co. KG (Eschborn, Germany). CTC was from Polysciences Europe GmbH (Eppelheim, Germany). DAPI was from Sigma‐Aldrich (Germany). Potassium phosphate, (NH_4_)_2_SO_4_, MgSO_4_, FeSO_4_, and glucose were from Merck (Darmstadt, Germany). Brain Heart Infusion (BHI) medium was purchased from Merck (Darmstadt, Germany). DNase was purchased from Peqlab (Erlangen, Germany). For FISH, 16S rRNA‐targeted oligonucleotide probe Stemal (purchased from Eurofins) for *S. maltophilia* was utilized. The probe was labeled with ATTO550 at the sequence (5'–3') of the probe sequence (GTCGTCCAGTATCCACTGC). For *P. aeruginosa*, 16S rRNA‐targeted oligonucleotide probe PseaerB (purchased from Eurofins) was utilized. The probe was labeled with AT488 at the sequence (5'–3') of the probe sequence (TCTCGGCCTTGAAACCCC). AxioImage M2 system equipped with an Apotome (Carl Zeiss, Oberkochen, Germany) was used for fluorescence microscopy. FluoSpheres carboxylate‐modified microspheres, 1.0 µm, red fluorescent (580/605), 2% solids were purchased from ThermoFisher Scientific (Germany).

*Preparation of Patterned SLIPS*: Patterned superhydrophobic–hydrophilic glass slides were dipped into 70% ethanol for 10 min. After drying, the slides were dipped into deionized (DI) water to form droplets in hydrophilic regions, which were separated by superhydrophobic regions without water. After that, a thin layer of Krytox GPL 103 was spread over the surface to cover the whole slides but only penetrated into the hydrophobic regions. The extra Krytox liquid was removed by dipping the slides into water for 20 times and flushing with a stream of water for 30 s.

*Biofilm Formation on Patterned SLIPS*: Liquid cultures of *Pseudomonas aeruginosa* (PA30, PA49), isolated from environmental wastewater,^29^ *Stenotrophomonas maltophilia* DSM50170 (*S. maltophilia*) and *Staphylococcus aureus* DSM20231 (*S. aureus*) were prepared in diluted BHI medium (1:4 BHI:water) with OD_600_ = 0.1. To form biofilms, patterned SLIPS slides were immersed into bacterial suspension and incubated for determined times at 37 °C with 50 rpm shaking for a better nutrient distribution. In parallel, biofilms were also cultivated under static conditions without shaking. Biofilm bridging did also occurred under these conditions in a comparable way (Supporting Information). The medium was refreshed every 24 h. Slides were washed with buffer (5 × 10^−3^ m magnesium acetate, 10 × 10^−3^ m Tris‐base, pH = 8) after incubation of defined periods of time. To stain with CTC and DAPI, slides were first immersed into a CTC solution (4 × 10^−3^ m freshly given to the medium) for 3 h at 37 °C with 50 rpm shaking or without shaking, according to previous incubation condition. After that, the slides were put into DAPI solution (1 µg mL^−1^, water solution) and incubated for 10 min. Epifluorescence microscopy with AxioImage M2 imaging system was applied to observe and take images of biofilms and bridges. To quantify the biofilm bridges, the number of bridges per area (cm^2^), width of bridges in the middle, and length of bridges in images were counted and measured with ImageJ software. At least 20 images for each sample were taken with the microscope and five samples for each bacterial species for statistics were also taken.

For bacterial bridges analysis, 1000‐folds magnification and Z‐stacks were applied to obtain the images of stained biofilm bridges of *P. aeruginosa* PA 49.

To test the nutrient effect on bridges formation, basal medium 2 (BM2; 62 × 10^−3^ m potassium phosphate, 7 × 10^−3^ m (NH_4_)_2_SO_4_, 2 × 10^−3^ m MgSO_4_, 10 × 10^−6^ m FeSO_4_, and 0.4% glucose) was used in the incubation of *P. aeruginosa* PA49, missing ether MgSO_4,_ FeSO_4,_ or glucose from the medium. To investigate the growth of bridges, patterned SLIPS incubated in *P. aeruginosa* PA 49 BM2 medium suspension were removed out from bacterial suspension after 0, 1, 2, 3, 4, 5, 6, and 24 h to stain with DAPI.

To test the effect of DNase on bridges formation, *P. aeruginosa* PA30, PA49, and *S. maltophilia* DSM50170 were incubated with SLIPS slides in BHI medium (1:4 water dilution) for 24 h with 50 rpm shaking at 37 °C. DNase (2 and 4 U mL^−1^) was added into the solution from the start for some samples. SLIPS samples were removed from bacterial suspension after 24 h incubation, and stained with CTC and DAPI.

To stain the biofilm bridges with 1 µm carboxylate‐modified microspheres loaded with red dyes, the patterned SLIPS were incubated in BM2 medium with or without *P. aeruginosa* PA49 (optical density of 600 nm was 0.1) for 24 h at 37 °C. The samples were then stained with DAPI for 10 min as described above. After washing with DI water for three times, 10 mL of the solution of the microbeads (10^5^ mL^−1^, water solution) was added to the sample, followed by 10 min incubation. The samples were taken out of the medium and imaged by epi‐fluorescence microscopy.

*Formation of Biofilm Bridges of Multiple Species Bacteria on Patterned SLIPS and FISH Staining*: Mixture suspension of *P. aeruginosa* PA 49/*S. maltophilia* (DSM50170) (v/v = 1:1) and *P. aeruginosa* PA 30/*S. maltophilia* (DSM50170) (v/v = 1:1) were prepared with initial concentration of each species suspension was all the same (OD_600_ = 0.1). SLIPS samples were incubated in bacteria suspension for 24 h with 50 rpm shaking at 37 °C. Then samples were removed from the solution, washed, fixed, and treated with FISH hybridization buffer. The samples were fixed with 4% paraformaldehyde solution (in phosphate buffered saline buffer, pH = 7.4) for 1 h at room temperature. Then samples were immersed into lysozyme solution (70 000 U mL^−1^ in Tris‐HCL pH = 7.5) for 10 min at 37 °C. After the fixation and permeabilization, samples were adjusted in hybridization buffer with adequate formamide concentration (0.9 m NaCl, 20 × 10^−3^ m Tris‐HCL, pH = 7.5, 0.01% sodium dodecyl sulfate, 30% formamide) for 10 min at 46 °C. Samples were immersed in 500 µL of the same solution previously mixed with FISH probes (purchased from Eurofins) for 1.5–3 h at 46 °C. The concentration of probes was 6 ng oligonucleolide µL^−1^. Finally, the samples were immersed in cell wash buffer for 10 min at 46 °C. After washing with wash buffer again, the samples were imaged by epi‐fluorescence microscopy.

*Quantification of Biofilm Occupation and Bridges*: As described in previous study,^13^ DAPI staining presenting DNA (biomass) in biofilm was quantified as biofilm occupation. Binary images were produced using ImageJ software and were inverted to make the biofilms show black or gray color. Then threshold‐adjusting option of ImageJ software was used to choose biofilm occupation area (DAPI staining). To mak sure all DAPI staining area were chosen for further calculation; the threshold was adjusted to the level, which was able to include all pixels appearing gray or black (not white). Then the biofilm occupation is

(1) $$\mathrm{biofilm}\;\mathrm{occupation}\;\left(\%\right)\;=\;\frac{\mathrm{area}\;\mathrm{of}\;\mathrm{DAPI}\;\mathrm{staining}\;\mathrm{in}\;\mathrm{one}\;\mathrm{hydrophilic}\;\mathrm{square}}{\mathrm{total}\;\mathrm{area}\;\mathrm{of}\;\mathrm{one}\;\mathrm{hydrophilic}\;\mathrm{square}}\;\times 100\%$$

Number of bridges on SLIPS was visually counted with fluorescence images. Distance between two edges of middle part of bridge was calculated as width of bridges with the distance measuring option of ImageJ software. Distance from one end to another end of bridges in hydrophilic spots was calculated as length of bridges with the distance measuring option of ImageJ software. At least ten images were analyzed for each sample.

*Statistical Analysis*: All data were presented as mean ± SD. Experiments were at least repeated twice individually using *n* ≥ 5 repetitions. All data were analyzed with two‐sided Student's *t*‐test using OriginPro (OriginLab Corporation) software. Data with *p*‐values < 0.05 were considered statistically significant.

## Conflict of Interest

The authors declare no conflict of interest.

## Supporting information

Supplementary
