# Supplementary material for: Biofilm Bridges Forming Structural Networks on Patterned Lubricant‐Infused Surfaces
Source: Adv Sci (Weinh). 2019 May 8;6(13):1900519. doi: 10.1002/advs.201900519 (PMC6662098; doi:10.1002/advs.201900519)
Supplement: Supplementary file 1 — Supplementary [file ADVS-6-1900519-s001.pdf]

Copyright WILEY-VCH Verlag GmbH & Co. KGaA, 69469 Weinheim, Germany, 2019.

ADVANCED SCIENCE
Open Access

Supporting Information

for *Adv. Sci.,* DOI: 10.1002/advs.201900519

Biofilm Bridges Forming Structural Networks on Patterned Lubricant-Infused Surfaces

Wenxi Lei, Julia Bruchmann, Jan Lars Rüping, Pavel A. Levkin, and Thomas Schwartz**

Supporting Information

Biofilm bridges forming structural networks on patterned lubricant-infused surfaces

W. Lei, J. Bruchmann, J. Rüping, P. A. Levkin, and T. Schwartz**

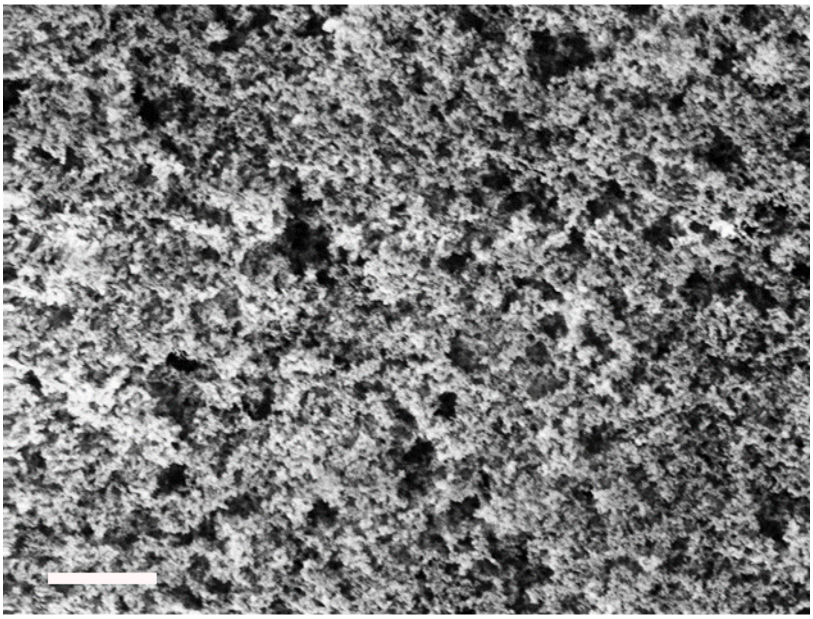

Figure S1. SEM image of the substrate of patterned SLIPS without lubricant. The scale bar: 1 µm.

Surfaces without lubricant were prepared. Prior to SEM measurements, samples were sputtered with a 10 nm gold layer using a Cressington 108 auto sputter coater. LEO 1530 Gemini scanning electron microscope (Zeiss, Germany) was used to take images of the substrate of patterned SLIPS. The SEM image shows the porous structure of the surface, which is required to lock the lubricant for SLIPS preparation.

Table S1 Water contact angle and sliding angle of different area on patterned SLIPS.

	Static water contact angle	Advancing water contact angle	Receding water contact angle	Sliding angle for water
Hydrophilic area	0 °	-	-	-
Hydrophobic area	156.5° ± 3°	162.6° ± 4°	152.5° ± 4°	1.9° ± 0.4°
SLIPS area	100.3° ± 1°	100.4° ± 5°	95.5° ± 2°	1.6° ± 0.2°

We measured the water contact angle and sliding angle with DSA 25 contact angle goniometer (Krüss, Germany) using the sessile drop technique. Advancing contact angles were obtained by measuring the angle while the liquid was slowly added at a rate of 0.1 mL s^{-1} from a ~4 μL droplet to 14 μL in contact with the sample and a micrometer syringe. Receding contact angles were obtained with liquid slowly retracting at a rate of 0.1 mL s^{-1} from a ~14 μL droplet to 4 μL. Sliding angles were measured by using the tilting option with the rate of 60° min^{-1}. The table shows small sliding angles of the lubricant infused surfaces, while the advancing water contact angles and receding water contact angles of the surface were smaller than those of the hydrophobic area, indicating the hydrophobic surfaces turned into slippery surfaces after the spread of lubricant.

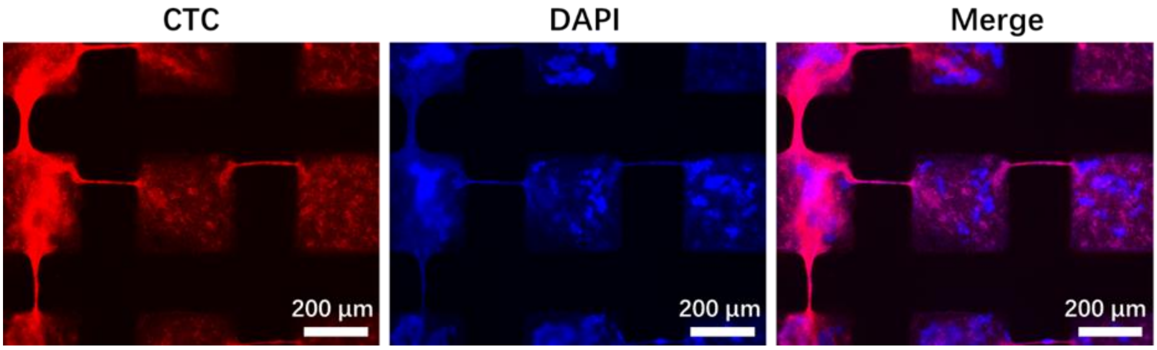

Figure S2. Fluorescence microscope images of biofilms of *P. aeruginosa* PA 49 on patterned SLIPS after 1 day incubation in BM2 medium under static condition (without shaking). Biofilms were stained with CTC for 3 h then with DAPI for 10 min before images were produced. Red color represents active bacteria from CTC staining and blue color represents DNA (external+inside of bacteria). The microscope observations were completed by ImageJ software.

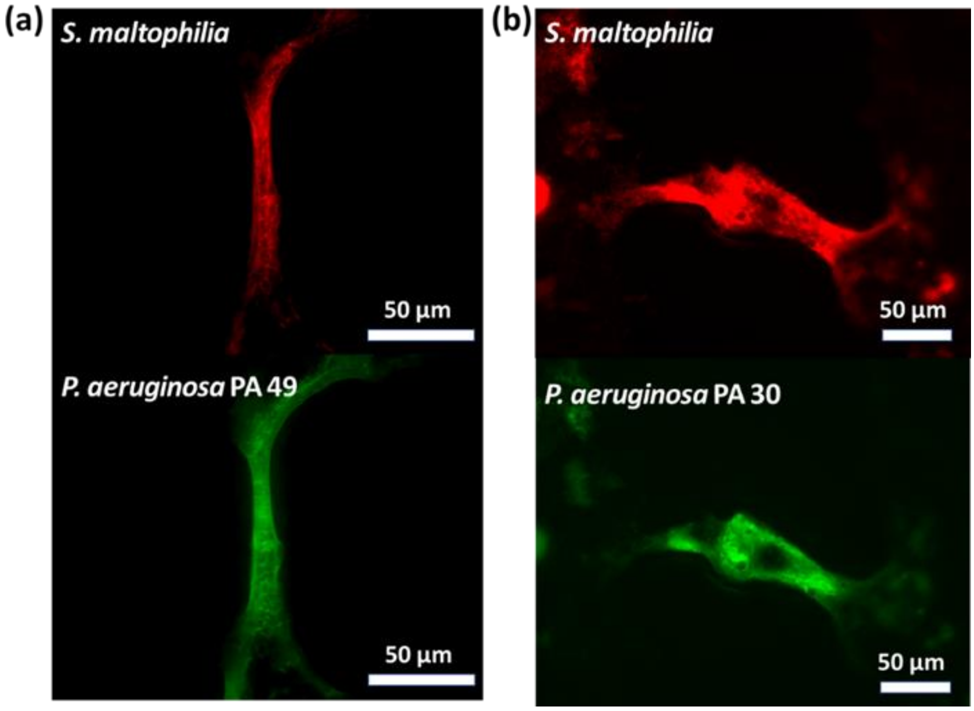

Figure S3. Images of mixed species biofilm bridges after FISH staining (individual fluorescence channel). (a) A single biofilm bridge where red fluorescence is from *S. maltophilia* (top) and green fluorescence comes from *P. aeruginosa* PA 49 (bottom). (b) Biofilm bridges of a mixed population of PA 30 (green fluorescence, bottom) and *S.maltopohilia* (red fluorescence, top). Patterned SLIPS slides were incubated with the bacteria mixture solution for 24 h, followed by FISH staining.

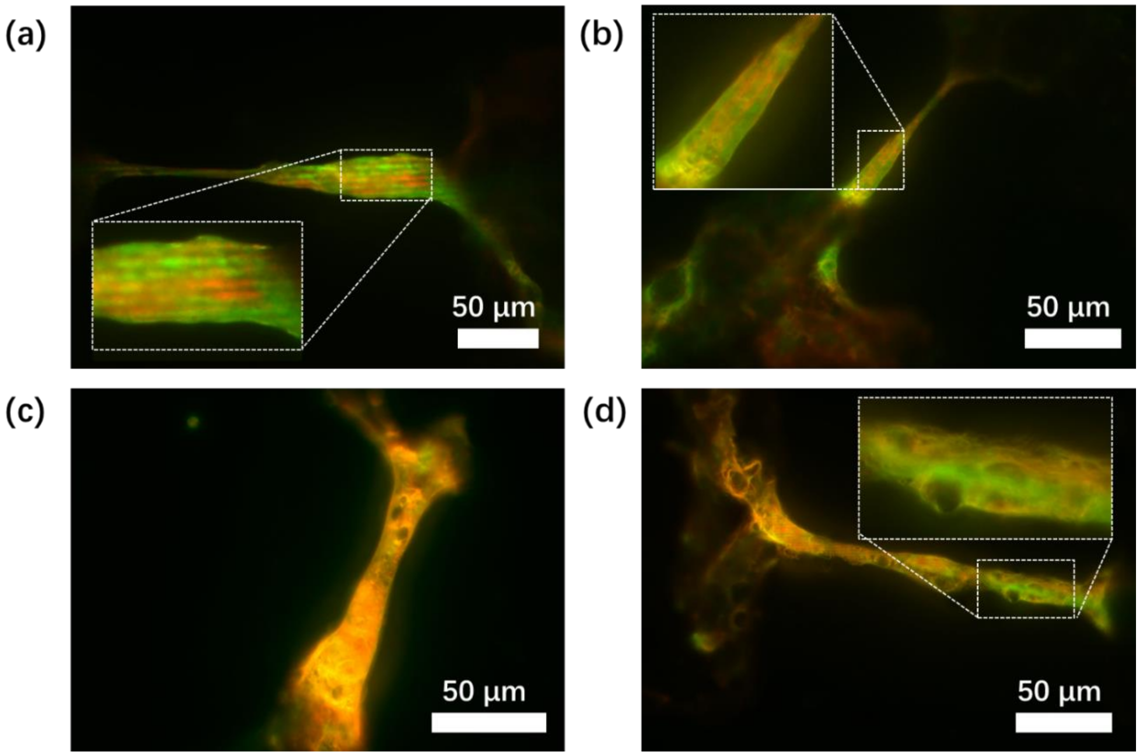

Figure S4. Images of mixed species biofilm bridges after FISH staining. (a) A single biofilm bridge where red fluorescence is from *S. maltophilia* and green fluorescence comes from *P. aeruginosa* PA 30. (b)(c)(d) Biofilm bridges of a mixed population of *P. aeruginosa* PA 49 (green fluorescence) and *S. maltophilia* (red fluorescence). Patterned SLIPS slides were incubated with the bacteria mixture solution for 24 h, followed by FISH staining.

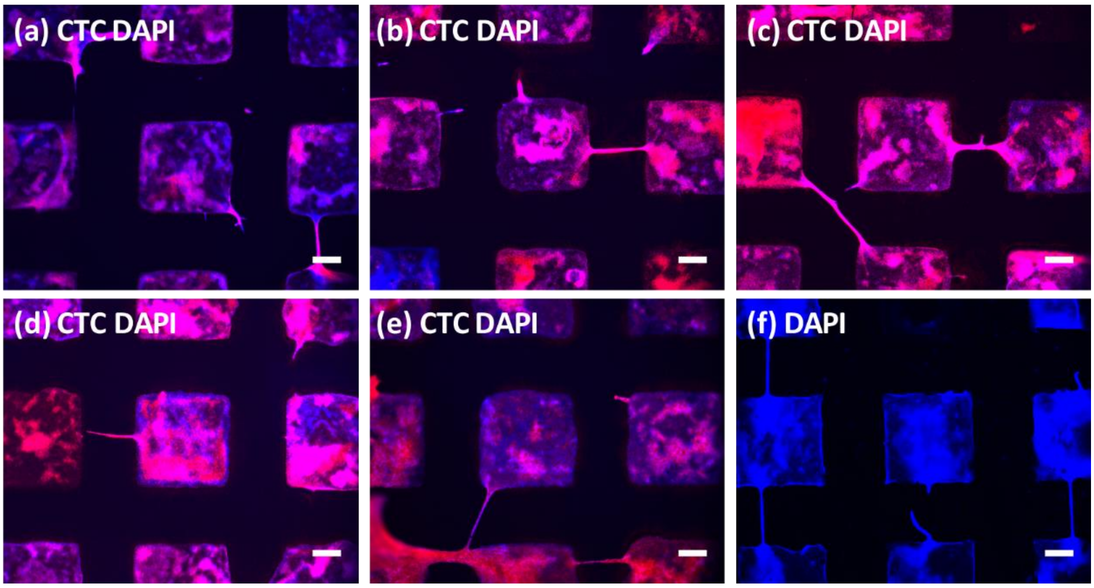

Figure S5. Fluorescence microscope images of biofilm bridges of *P. aeruginosa* PA 49 of different length on patterned SLIPS after 1 day incubation in BM2 medium. Biofilms were stained with CTC for 3 h then with DAPI for 10 min before images were produced. Red color represents active bacteria from CTC staining and blue color represents DNA (external + inside of bacteria). The microscope observations were completed by ImageJ software. The scale bar is 100 µm.

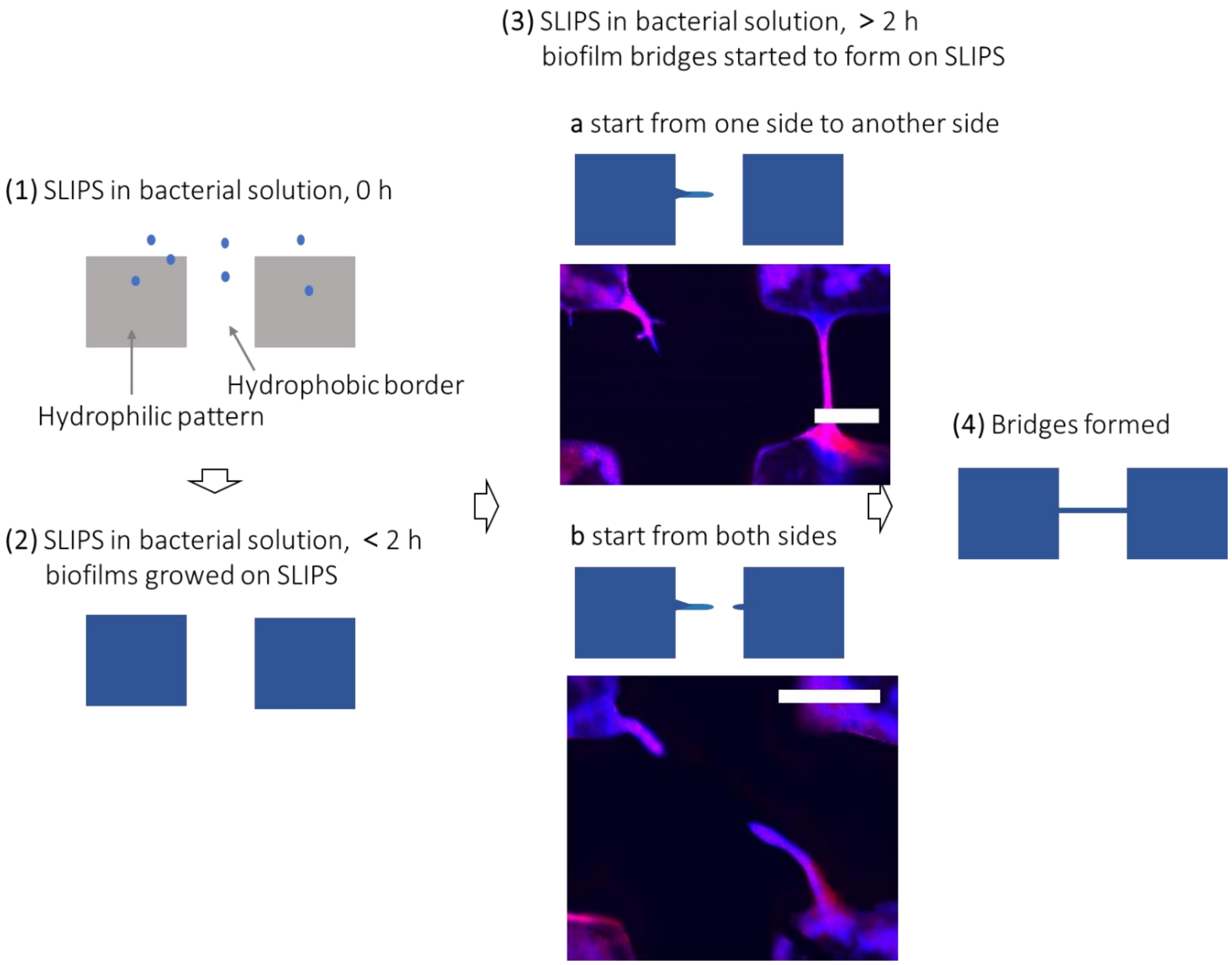

Figure S6. Scheme of biofilm bridge formation. Fluorescence images inserted were of bridges after 24 h incubation of patterned SLIPS with *P. aeruginosa* PA49 in BM2 medium, and bridges were stained with DAPI and CTC. The scale bars: 100 μm.

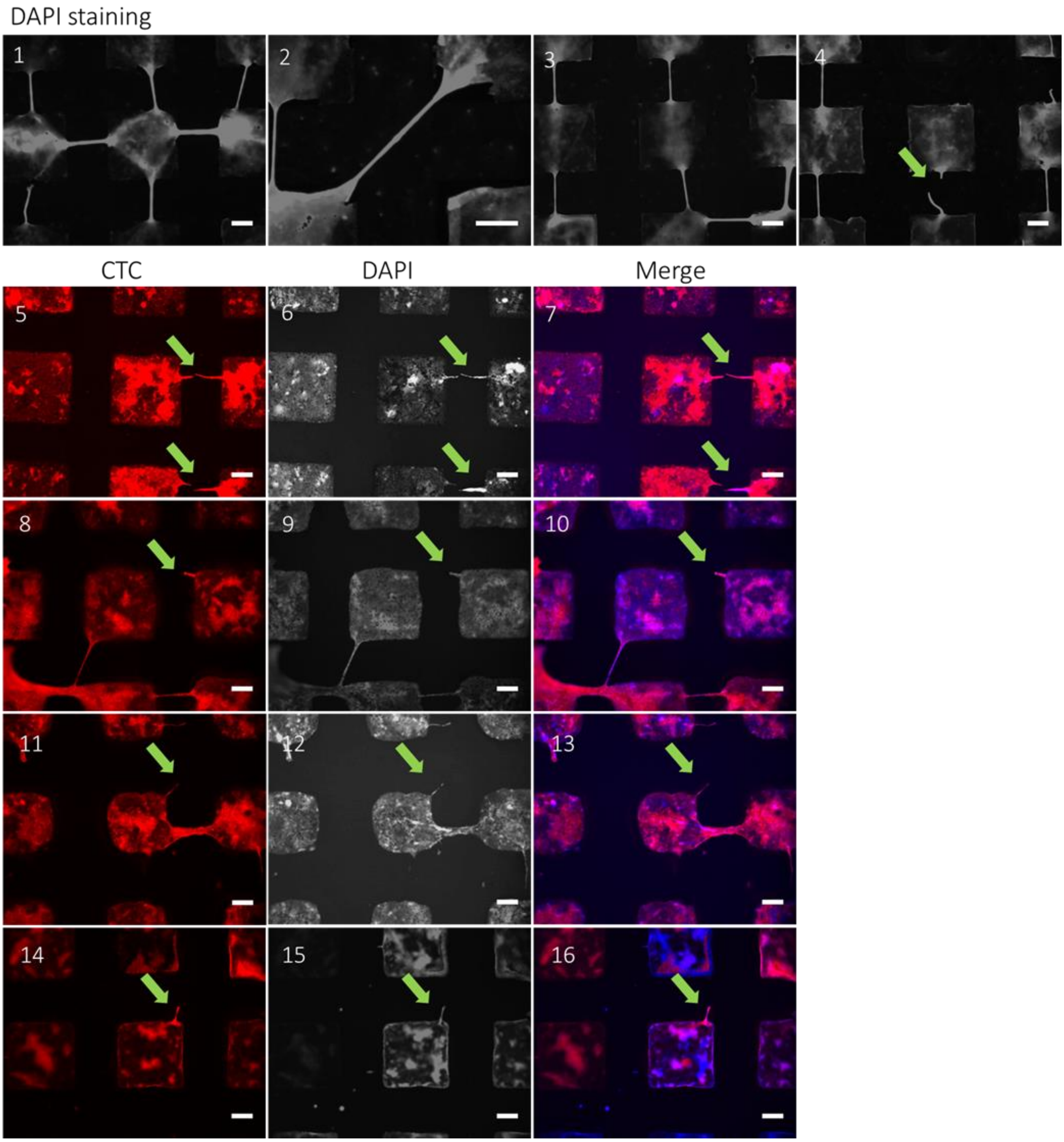

DAPI staining
1
2
3
4
CTC
DAPI
Merge
5
6
7
8
9
10
11
12
13
14
15
16

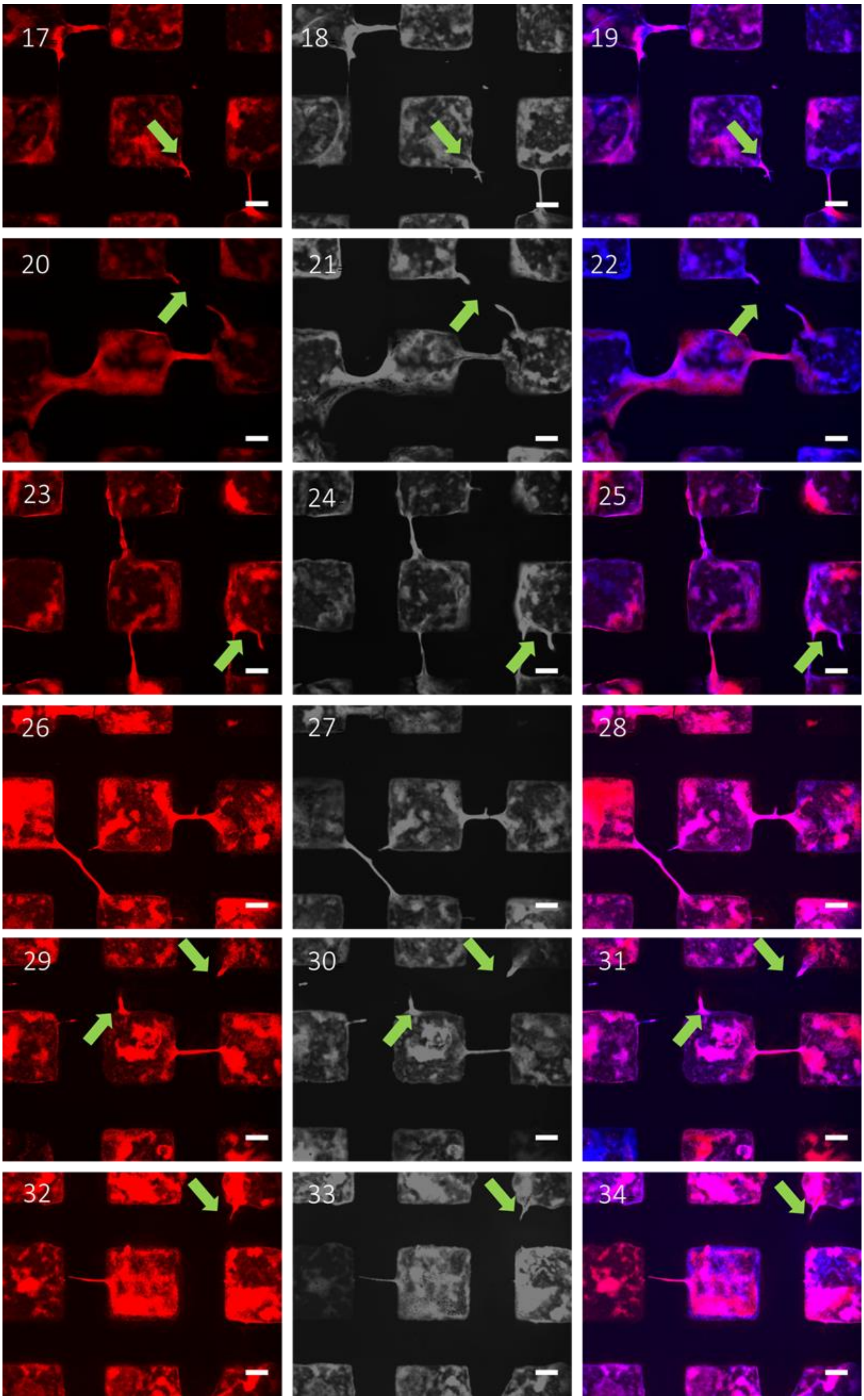

Figure S7. Fluorescence microscope images of biofilm bridges of *P. aeruginosa* PA 49 on patterned SLIPS after 1 day incubation in BM2 medium. Biofilms were stained with CTC for 3 h then with DAPI for 10 min before images were produced (some of them were only stained with DAPI, we sued white color to show the DAPI staining). Red color represents active bacteria from CTC staining and blue color represents DNA (external + inside of bacteria). The microscope observations were completed by ImageJ software. The scale bar is 100 µm.
